# Neural correlates of tactile simultaneity judgement: a functional magnetic resonance imaging study

**DOI:** 10.1038/s41598-019-54323-7

**Published:** 2019-12-20

**Authors:** Takahiro Kimura, Hiroshi Kadota, Tsuyoshi Kuroda, Tomomi D. Funai, Makoto Iwata, Takanori Kochiyama, Makoto Miyazaki

**Affiliations:** 1 Institute of Liberal Arts and Science, Kanazawa University, Kakuma-machi, Kanazawa-shi, Ishikawa 920-1192 Japan; 2 Research Institute, Kochi University of Technology, 185 Miyanokuchi, Tosayamada, Kami-shi, Kochi 782-8502 Japan; 3 School of Information, Kochi University of Technology, 185 Miyanokuchi, Tosayamada, Kami-shi, Kochi 782-8502 Japan; 4 Faculty of Informatics, Shizuoka University, 3-5-1 Johoku Naka-ku, Hamamatsu-shi, Shizuoka 432-8011 Japan; 5 Research Institute of Time Study, Yamaguchi University, 1677-1 Yoshida, Yamaguchi, 753-8511 Japan; 6 ATR Brain Activity Imaging Center, 2-2-2 Hikaridai Seika-cho, Sorakugun, Kyoto 619-0288 Japan

**Keywords:** Perception, Sensory processing

## Abstract

Simultaneity judgement (SJ) is a temporal discrimination task in which the targets span an ultimately short time range (zero or not). Psychophysical studies suggest that SJ is adequate to probe the perceptual components of human time processing in pure form. Thus far, time-relevant neural correlates for tactile SJ are unclear. We performed functional magnetic resonance imaging (fMRI) to investigate the neural correlates of tactile SJ using tactile number judgement as a time-irrelevant control task. As our main result, we demonstrated that the right inferior parietal lobule (IPL) is an SJ-specific region. The right IPL was detected by both parametric and non-parametric statistical analyses, and its activation intensity fulfilled a strict statistical criterion. In addition, we observed that some left-dominant regions (e.g., the striatum) were specifically activated by successive stimuli during SJ. Meanwhile, no region was specifically activated by simultaneous stimuli during SJ. Accordingly, we infer that the neural process for tactile SJ is as follows: the striatum estimates the time interval between tactile stimuli; based on this interval, the right IPL discriminates the successiveness or simultaneity of the stimuli. Moreover, taking detailed behavioural results into account, we further discuss possible concurrent or alternative mechanisms that can explain the fMRI results.

## Introduction

Time impacts our life and behaviours across various time scales, such as from days (i.e., circadian rhythms) to milliseconds (e.g., motor controls)^1^, and different time ranges engage different brain regions (e.g., circadian rhythm: suprachiasmatic nucleus^2^, motor control: cerebellum^3^). Numerous neuroimaging studies have reported brain regions underlying the perception of time intervals across a range of a few seconds to several hundred milliseconds (see reviews^4–7^). However, reports regarding brain regions involved in the perception of shorter time ranges of less than 0.1 s are still scarce. The perception of 10-millisecond ranges has been investigated using temporal order judgement (TOJ)^8–10^ and simultaneity judgement (SJ)^9,11,12^ tasks. In both tasks, participants receive two sensory stimuli. In TOJ, the participants then judge which stimulus is earlier (or later). In SJ, the participants then judge whether the stimuli are simultaneous. Therefore, SJ is a task to probe brain function underlying the discrimination of ultimately short times (zero or a short nonzero interval).

Psychophysical studies have indicated that participants can correctly discriminate stimuli with shorter stimulus intervals in SJ than in TOJ, even when using a pair of audio^13^, visual^14^, or tactile^15^ stimuli. That is, when participants receive two stimuli separated by a very short interval, they can discriminate whether the stimuli are simultaneous or successive but cannot identify which stimulus was earlier or later. Based on this observation, it was proposed that the discrimination of simultaneity/successiveness of sensory stimuli is a necessary but not sufficient condition for the identification of the temporal order of the stimuli^16,17^. This conditional relationship suggests that the processes for SJ are included in those for TOJ but that TOJ requires additional processes.

In our previous functional magnetic imaging (fMRI) study^9^, we directly compared the neural correlates of tactile TOJ with those of SJ. Compared to SJ, TOJ more strongly activated the left-dominant multiple regions: the left posterior parietal cortex (PPC), the left-ventral and bilateral-dorsal premotor cortices (PMC), and the bilateral thalamus (also see references^18,19^). Furthermore, an SJ-specific region was also found. Compared to TOJ, SJ more strongly activated the left posterior insula. Based on this observation, we proposed that the posterior insula acts as a detector^14,20^ or comparator^21^ of tactile simultaneity.

Previous neuroimaging studies have also reported that the insula is activated in SJ tasks using other sensory modalities^11,12^, although the anteroposterior positions and left-right dominance of the activated areas vary. Bushara *et al*.^11^ investigated neural correlates of audio-visual SJ using a colour judgement with attending to audio stimuli as a control task. They reported that the right anterior insula exhibited the greatest SJ-specific activity. Lux *et al*.^12^ conducted fMRI on participants engaged in a visual SJ and an orientation judgement, reporting that the right parietal insula was specifically activated in SJ.

However, in these studies^11,12^, SJ-specific activity was observed not only in the insula but also in other regions of the brain. In audio-visual SJ^11^, SJ-specific activity was observed in the right inferior parietal lobule (IPL), inferior frontal gyrus (IFG), and left cerebellum. In visual SJ^12^, SJ-specific activity was also detected in the left temporal-parietal junction (TPJ), IFG, middle frontal gyrus (MFG), and superior temporal gyrus (STG). Notably, these SJ-specific regions were detected using time-irrelevant control tasks (colour or orientation judgement). In contrast, in our previous study^9^, SJ-specific insula activity was detected using a time-relevant contrastive task (i.e., TOJ), which allows the subtraction of common time-relevant neural activities between SJ and TOJ. Thus, our previous study^9^ inevitably could not detect some time-relevant neural correlates of tactile SJ.

In the present study, we conducted fMRI to reveal time-relevant neural correlates of tactile SJ using a time-irrelevant control task. This research is necessary to identify neural correlates of tactile time perception of ten-millisecond ranges in pure form. As described above, psychophysical studies^16,17^ suggest that TOJ involves more processes than SJ. Indeed, some psychophysical effects are specifically observed in TOJ but not in SJ. The crossed-hand deficit^22,23^ is a striking example. Crossing the arms causes misreporting of the temporal order of two tactile stimuli delivered one to each hand. This deficit suggests that the brain takes the spatial location of the hands into account during tactile TOJ. In contrast, crossing the arms has no effect on tactile SJ^15,24^. The crossed-hand deficit provides us with implications for the spatiotemporal representation of the body in the brain. However, this phenomenon also indicates that, if the focus is only on “time”, SJ is clearly a better choice than TOJ for identifying the neural correlates.

What brain regions are involved in the time-relevant processes of tactile SJ? Psychophysical studies suggest that SJ-related regions may differ among the sensory modalities^17^. Therefore, we cannot always deduce the neural correlates of tactile SJ from those of audio-visual^11^ or visual^12^ SJs. However, considering previous fMRI studies on tactile TOJs, we can identify possible SJ-related regions that went undetected in the SJ > TOJ contrast in our previous study. Takahashi *et al*.^10^ investigated neural correlates of tactile TOJ using a “greater/smaller” type of tactile number judgement (NJ) for a time-irrelevant control task. The TOJ > NJ contrast showed greater activity in the bilateral frontal and parieto-temporal regions (e.g., PMCs, TPJs). Meanwhile, the TOJ > SJ contrast in our previous study^9^ revealed increased activity in the left-dominant frontal and parietal regions. The regions that were observed in the TOJ > NJ contrast but not in the TOJ > SJ contrast should include SJ-related activity that was cancelled in the SJ > TOJ contrast. Accordingly, we can expect that the SJ-related activity may be found in some of the right frontal and parieto-temporal regions. Based on this hypothesis, we investigated the neural correlates of tactile SJ using the “same/different” type of tactile NJ for the control task.

## Results

**Figure 1:**
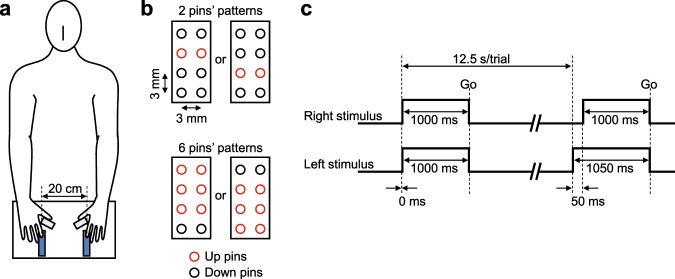
Experimental setups and designs: (**a**) a participant with stimulus-response devices; (**b**) arrangements of the pins for stimulation; (**c**) stimulation sequence and timing.

During fMRI scanning, the participants (*n* = 32) performed a tactile SJ and a same/different type of tactile NJ. For each trial, the participants received two tactile stimuli across the ventral pads of both index fingers (Fig. 1a). The tactile stimuli were presented using a pair of non-magnetic 2 × 4-pin Braille stimulators (Fig. 1a,b). The stimulus onset asynchronies (SOAs) between the left and right stimuli were −50 ms (left earlier), 0 ms (simultaneous), or +50 ms (right earlier). The appearance ratio of these SOAs was 1:2:1. Therefore, simultaneous and successive stimuli each appeared in 50% of trials. The number of driven pins in each tactile stimulator was 2 or 6 (Fig. 1b), resulting in a difference in the number of pins of −4 (left greater), 0 (same), or +4 (right greater). The appearance ratio of the number differences was 1:2:1. Therefore, the same and different number of stimuli each appeared in 50% of trials. The stimulus conditions (timings × numbers) were presented in pseudo-random orders.

In SJ, the participants judged whether the onsets of left and right stimuli were simultaneous. In NJ, the participants judged whether the numbers of pins in the left and right stimuli were the same. Each participant performed 2 sessions (32 trials/session) of SJ and 2 sessions of NJ (i.e., 4 sessions in total).

### Behavioural results

#### Comparison between tasks

Table 1 shows the accuracy rates and reaction times for SJ and NJ. In the accuracy rates, there was no difference between SJ and NJ [*t*(31) = 0.13, *p* = 0.90, Cohen’s *d* = 0.02, paired *t*-test]. The reaction time was significantly longer for NJ than for SJ [*t*(31) = 2.98, *p* = 0.006, *d* = 0.53], which is not a problem for interpreting regions associated with SJ in the SJ > NJ contrast (e.g., Davis *et al*.^8^).

**Table 1:** Accuracy rates and reaction times [mean (standard deviation)] across the participants for simultaneity judgement (SJ) and number judgement (NJ).

	Tasks	
	SJ	NJ
Accuracy rate	0.86 (0.10)	0.85 (0.10)
Reaction time (ms)	830.5 (303.1)	920.6 (262.3)

#### Comparisons among tasks and stimulation types

Table 2 shows the accuracy rates and reaction times calculated for each task (SJ/NJ) and each stimulation type (same/different).

**Table 2:** Accuracy rates and reaction times [mean (standard deviation)] across the participants for task (SJ/NJ) × stimulation type (same/different).

	Stimulation type	Tasks	
		SJ	NJ
Accuracy rate	same	0.93 (0.10)	0.88 (0.12)
	different	0.78 (0.17)	0.83 (0.13)
Reaction time (ms)	same	819.1 (346.1)	941.7 (278.7)
	different	841.8 (271.3)	899.6 (274.3)

SJ: simultaneity judgement; NJ: number judgement.

A two-way repeated-measures analysis of variance (ANOVA) (2 tasks × 2 stimulation types) on the accuracy rates revealed no significant main effect of the task [*F*(1, 31) = 0.02, *p* = 0.90, *η*_*p*_^2^ = 0.0006], further confirming that there was no difference in the accuracy rates between SJ and NJ.

The ANOVA indicated a significant main effect of stimulation type [*F*(1, 31) = 21.93, *p* < 0.001, *η*_*p*_^2^ = 0.41] and a significant interaction between task and stimulation type [*F*(1, 31) = 5.23, *p* = 0.029, *η*_*p*_^2^ = 0.14]. The analyses of simple main effects for the interaction revealed that the effect of stimulation type was significant at SJ [*F*(1, 31) = 18.19, *p* < 0.001, *η*_*p*_^2^ = 0.37] but did not reach significance at NJ [*F*(1, 31) = 3.63, *p* = 0.07, *η*_*p*_^2^ = 0.095]. The effect of task was not significant for the stimulation type “same” [*F*(1, 31) = 3.25, *p* = 0.08, *η*_*p*_^2^ = 0.10] or for “different” [*F*(1, 31) = 1.43, *p* = 0.24, *η*_*p*_^2^ = 0.04]. These results indicate that the accuracy rate was higher when simultaneous stimuli were presented than when successive stimuli were presented in SJ. Notably, in the interaction contrasts among the tasks and stimulation types, we regressed out the correspondence differences in the accuracy rates among the conditions (for details, see fMRI data analysis in Methods).

A two-way repeated-measures ANOVA (2 tasks × 2 stimulation types) on the reaction times revealed a significant main effect of task [*F*(1, 31) = 8.88, *p* = 0.006, *η*_*p*_^2^ = 0.22]. The ANOVA did not reveal a significant main effect of stimulation type [*F*(1, 31) = 0.19, *p* = 0.67, *η*_*p*_^2^ = 0.006] or an interaction between task and stimulation type [*F*(1, 31) = 3.86, *p* = 0.059, *η*_*p*_^2^ = 0.11]. These results are consistent with that of the *t*-test between the tasks.

### fMRI results

We analysed the fMRI data using SPM12 (http://www.fil.ion.ucl.ac.uk/spm). Significantly activated voxels were identified using a threshold of *p* < 0.001 uncorrected at the voxel level and *p* < 0.05 family-wise error (FWE) corrected at the cluster level^25^.

Moreover, we also conducted a non-parametric statistical analysis with a statistical nonparametric mapping (SnPM) toolbox, SnPM13 (http://warwick.ac.uk/snpm). A recent study^26^ reported that false positive results may occur when cluster-level correction is used for multiple comparisons in standard parametric statistical methods such as statistical parametric mapping (SPM). Notably, SnPM was developed by an author of the critical report highlighting this issue^26^.

#### Contrasts between tasks

**Figure 2:**
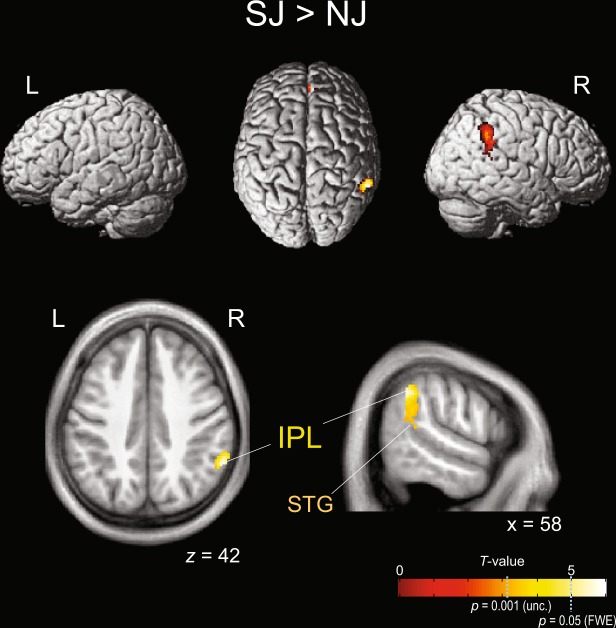
Brain regions that were more strongly activated during simultaneity judgement (SJ) than during number judgement (NJ) (*n = *32). Activated voxels were calculated with SPM12 using a threshold of *p* < 0.001 uncorrected at the voxel level (*T* > 3.16) and *p* < 0.05 FWE corrected at the cluster level. This SJ > NJ contrast was inclusively masked with the SJ > rest contrast (*p* < 0.05 uncorrected). In particular, the activation at the right IPL also exceeded the significance threshold of *p* < 0.05 FWE corrected at the voxel level (*T* > 5.00). IPL: inferior parietal lobule; STG: superior temporal gyrus.

Figure 2 and Table 3 show a region activated more strongly for SJ than for NJ as calculated with SPM12. The SJ > NJ contrast exhibited significantly increased activation in the right IPL, extending to the STG. Notably, the SJ-related activation at the right IPL also exceeded the significance threshold of *p* < 0.05 FWE corrected at the voxel level (the cluster size exceeding the threshold was 17 voxels). In this contrast, increased activation was also observed in the right anterior cingulate gyrus (ACG) and medial frontal gyrus (MedFG). Notably, the posterior insula was not detected in the SJ > NJ contrast.

**Table 3:** Activated clusters in the SJ > NJ contrast, calculated with SPM12 (*p* < 0.001 uncorrected at the voxel level; *p* < 0.05 FWE corrected at the cluster level).

Cluster #	Size (voxels)	MNI coordinate			*T*_124_	L/R	Region
		x	y	z			
1	296	58	−48	42	5.52*	R	IPL
		60	−44	28	3.70	R	SMG
		58	−42	16	3.39	R	STG
2	298	8	46	0	4.08	R	ACG/MedFG
		0	46	18	3.86		MedFG/ACG

This contrast was inclusively masked with the SJ > rest contrast (*p* < 0.05 uncorrected). *denotes that the activation level exceeded the threshold of *p* < 0.05 FWE corrected at the voxel level (*T* > 5.00). IPL: inferior parietal lobule; SMG: supramarginal gyrus; STG: superior temporal gyrus; ACG: anterior cingulate gyrus; MedFG: medial frontal gyrus.

Table 4 shows a region activated more strongly for SJ than for NJ as calculated with SnPM13 (also see Supplementary Fig. S1). The SJ > NJ contrast with SnPM exhibited significantly greater activation only in the right IPL. The peak activation in the right IPL also exceeded the significance threshold of *p* < 0.05 FWE corrected at the voxel level.

**Table 4:** Activated cluster in the SJ > NJ contrast, calculated with SnPM13 (*p* < 0.001 uncorrected at the voxel level; *p* < 0.05 FWE corrected at the cluster level).

Cluster #	Size (voxels)	MNI coordinates			*T*_30_	L/R	Region
		x	y	z			
1	193	56	−48	42	5.79*	R	IPL

In this contrast, the difference in reaction times between the tasks was regressed out as a covariate of no interest. *denotes that the activation level exceeded the threshold of *p* < 0.05 FWE corrected at the voxel level (*T* > 5.60). IPL: inferior parietal lobule.

Table 5 shows the brain regions that were activated more strongly in NJ than in SJ, calculated with SPM12. In the NJ > SJ contrast, significantly greater activation was observed in multiple frontal-parietal areas, including the bilateral ventral PMCs and PPCs, and the right dorsal PMC and postcentral gyrus/supramarginal gyrus (SMG). Similar results were obtained for the NJ > SJ contrast with SnPM13 (Table 6).

**Table 5:** Activated clusters in the NJ > SJ contrast, calculated with SPM12 (*p* < 0.001 uncorrected at the voxel level; *p* < 0.05 FWE corrected at the cluster level).

Cluster #	Size (voxels)	MNI coordinates			*T*_124_	L/R	Region
		x	y	z			
1	206	−50	4	26	5.42*	L	PreCG
		−44	4	26	5.23*	L	PreCG/IFG
2	340	46	−28	44	5.39*	R	IPL/PoCG
		56	−18	28	4.54	R	PoCG/SMG
3	111	50	10	26	4.76	R	IFG
4	199	−28	−58	44	4.37	L	IPL/AG
		−22	−68	46	3.30	L	SPL
5	167	32	−64	32	4.19	R	MOG
		30	−66	46	3.72	R	AG
6	152	34	−4	48	3.79	R	PreCG/MFG

This contrast was inclusively masked with the NJ > rest contrast (*p* < 0.05 uncorrected). * denotes that the activation level exceeded the threshold of *p* < 0.05 FWE corrected at the voxel level (*T* > 5.00). PreCG: precentral gyrus; IFG: inferior frontal gyrus; IPL: inferior parietal lobule; PoCG: postcentral gyrus; SMG: supramarginal gyrus; AG: angular gyrus; SPL: superior parietal lobule; MOG: middle occipital gyrus; MFG: middle frontal gyrus.

**Table 6:** Activated clusters in the NJ > SJ contrast, calculated with SnPM13 (*p* < 0.001 uncorrected at the voxel level; *p* < 0.05 FWE corrected at the cluster level).

Cluster #	Size (voxels)	MNI coordinates			*T*_30_	L/R	Region
		x	y	z			
1	284	26	−60	42	5.94*	R	AG
		32	−64	46	5.61	R	SPL/AG
2	398	48	−28	42	5.73*	R	PoCG/IPL
3	180	28	−6	50	5.49	R	PreCG
4	213	8	6	50	5.42	R	MedFG
		−8	10	44	5.34	L	MCG
		−8	−4	54	4.78	L	MedFG
5	148	52	8	28	5.19	R	IFG
		62	8	26	4.04	R	PreCG
6	189	−48	2	22	4.98	L	PreCG/IFG
7	253	−28	−58	44	4.86	L	IPL/AG
		−28	−68	28	3.84	L	MOG
8	183	38	−44	56	4.25	R	SPL/IPL

In this contrast, the difference in reaction times between the tasks was regressed out as a covariate of no interest. * denotes that the activation level exceeded the threshold of *p* < 0.05 FWE corrected at the voxel level (*T* > 5.64). AG: angular gyrus; SPL: superior parietal lobule; PoCG: postcentral gyrus; IPL: inferior parietal lobule; PreCG: precentral gyrus; MedFG: medial frontal gyrus; MCG: middle cingulate gyrus; IFG: inferior frontal gyrus; MOG: middle occipital gyrus.

#### Interaction contrasts: task (SJ/NJ) × stimulation type (same/different)

**Figure 3:**
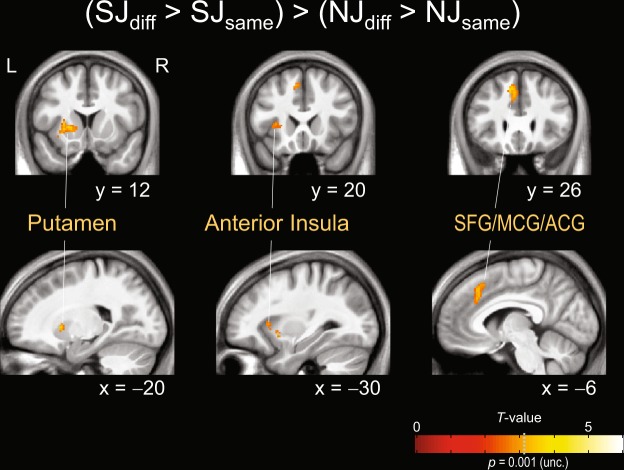
Brain regions that were specifically activated by successive stimuli during SJ (*n = *32). These activated regions were extracted using the (SJ_diff_ > SJ_same_) > (NJ_diff_ > NJ_same_) contrast calculated with SPM12. SJ_diff_: SJ with successive stimuli, SJ_same_: SJ with simultaneous stimuli, NJ_diff_: NJ with different-number stimuli, NJ_same_: NJ with same-number stimuli. Significantly activated voxels were identified using a threshold of *p* < 0.001 uncorrected at the voxel level (*T* > 3.16) and *p* < 0.05 FWE corrected at the cluster level. This contrast was inclusively masked with the SJ_diff_ > rest and SJ_diff_ > SJ_same_ contrasts (*p* < 0.05 uncorrected). SFG: superior frontal gyrus; MCG: middle cingulate gyrus; ACG: anterior cingulate gyrus.

Figure 3 and Table 7 show the interaction contrast of (SJ_diff_ > SJ_same_) > (NJ_diff_ > NJ_same_), calculated with SPM12. This contrast indicates the regions specifically activated when the tactile stimuli were successively presented during SJ. The results show greater activation in the left striatum (putamen, caudate) extended to the left anterior insula and in the left medial frontal areas including the ACG and middle cingulate gyrus (MCG) and the left superior frontal gyrus [SFG; corresponding to the pre-supplementary motor area (pre-SMA)].

**Table 7:** Activated clusters in the (SJ_diff_ > SJ_same_) > (NJ_diff_ > NJ_same_) contrast, calculated with SPM12 (*p* < 0.001 uncorrected at the voxel level; *p* < 0.05 FWE corrected at the cluster level).

Cluster #	Size (voxels)	MNI coordinates			*T*_124_	L/R	Region
		x	y	z			
1	235	−20	12	2	4.03	L	Putamen
		−30	20	6	3.52	L	Anterior Insula
		−16	16	4	3.38	L	Caudate
2	139	−6	26	40	3.81	L	SFG/MCG
		−8	30	28	3.42	L	ACG
		−10	18	50	3.39	L	SFG

This contrast was inclusively masked with the SJ_diff_ > rest and SJ_diff_ > SJ_same_ contrasts (*p* < 0.05 uncorrected). SFG: superior frontal gyrus; MCG: middle cingulate gyrus; ACG: anterior cingulate gyrus.

Regarding the other contrasts, no significant greater activity was detected in the (SJ_same_ > SJ_diff_) > (NJ_same_ > NJ_diff_), (NJ_diff_ > NJ_same_) > (SJ_diff_ > SJ_same_), nor (NJ_same_ > NJ_diff_) > (SJ_same_ > SJ_diff_) contrast. Note that appropriate masking was applied to each interaction contrast to avoid false activation due to deactivation in the contrastive conditions (for details, see fMRI data analysis in Methods).

Table 8 shows the (SJ_diff_ > SJ_same_) > (NJ_diff_ > NJ_same_) contrast, calculated with SnPM (also see Supplementary Fig. S2). The SnPM contrast exhibited increased activity in similar regions, as shown in the SPM contrast (Table 7). Additionally, in this contrast, the medial frontal activity extended to the right hemisphere, and significantly increased activity was also observed in the right anterior insula and IFG.

**Table 8:** Activated clusters in the (SJ_diff_ > SJ_same_) > (NJ_diff_ > NJ_same_) contrast, calculated with SnPM13 (*p* < 0.001 uncorrected at the voxel level; *p* < 0.05 FWE corrected at the cluster level).

Cluster #	Size (voxels)	MNI coordinates			*T*_30_	L/R	Region
		x	y	z			
1	301	26	24	−8	5.10	R	Anterior Insula
		50	20	6	4.38	R	IFG
2	596	−10	18	50	4.97	L	SFG
		−6	28	32	4.76	L	ACG/MCG
		8	32	30	4.36	R	ACG
		4	16	48	4.01	R	SFG
3	354	−22	12	2	4.93	L	Putamen
		−28	22	6	4.43	L	Anterior Insula
		−16	16	4	3.75	L	Caudate

In this contrast, the difference in accuracy rates among the conditions was regressed out as a covariate of no interest. IFG: inferior frontal gyrus; SFG: superior frontal gyrus; ACG: anterior cingulate gyrus; MCG: middle cingulate gyrus.

## Discussion

In the present study, we conducted fMRI to investigate the time-relevant neural correlates of tactile SJ using tactile NJ as a time-irrelevant control task. We also detected NJ-specific regions, including bilateral frontal-parietal areas, which is generally consistent with number-related areas in previous reports^27–30^. In the following sections, we discuss our results with a focus on SJ-related regions.

### SJ-specific regions

In the introduction, based on the comparison of previous fMRI studies^9,10^, we inferred that the time-relevant neural activity of tactile SJ may be detected in some of the right frontal and parieto-temporal regions. Indeed, the SJ > NJ contrast with SPM revealed SJ-specific activity in the right parieto-temporal region (i.e., the right IPL, SMG and STG). This region is also called the TPJ. In this region, the activity of the right IPL fulfilled the statistical criterion with the FWE correction not only at the cluster level but also at the voxel level. Moreover, the right IPL was detected not only by using a standard parametric statistical method (SPM) but also by using a novel non-parametric statistical method (SnPM). Our results reliably identify the right IPL as a time-relevant neural correlate of tactile SJ. In this section, we focus the remaining discussion on the right IPL.

The right IPL was suggested to be involved in TOJs by previous lesion and transcranial magnetic stimulation (TMS) studies. Lesion studies have reported that the perception of temporal order for separate visual events is impaired by right parietal damage^31,32^, in which the involvement of the right IPL was emphasized^31^. Woo *et al*.^33^ reported that TMS over the right PPC [mainly Brodmann area 40^34^, i.e., IPL] delayed the detection of a stimulus presented in the contralateral visual field. However, recent fMRI studies have demonstrated TOJ-specific activity not in the right IPL but rather in the left IPL^8,9^. In our previous fMRI study^9^, tactile TOJ more strongly activated the left PPC (IPL and superior parietal lobule) compared with tactile SJ, but not the right PPC. Additionally, in visual TOJ^8^, TOJ-specific activity was consistently observed in the left TPJ independent of the control tasks. Conflicting results regarding laterality between lesion/TMS studies and fMRI studies can be explained by the results of the present study, as discussed below.

Several psychophysical studies^16,17,35,36^ have proposed that the processes for SJ are included in those for TOJ but that TOJ requires further additional processes. This proposal suggests that the neural correlates of SJ are included in those of TOJ. Accordingly, the right IPL should be activated in TOJ as well as in SJ. Indeed, in a previous fMRI study^10^, tactile TOJ induced stronger activity in a similar right parietal region. Therefore, lesions and TMS of the right parietal region necessarily impair TOJs. Meanwhile, TOJ-specific processes (e.g., determination of the temporal order of stimuli) should more strongly activate TOJ-related regions, such as the left PPC^9^.

On the other hand, the right IPL was not observed as a neural correlate of visual SJ in a classic fMRI study^12^. Lux *et al*.^12^ reported neural correlates of visual SJ in the left TPJ, IFG, MFG and STG and the right parietal insula. Thus, visual SJ activated the left IPL (included in TPJ) but not the right IPL. However, a recent fMRI study^37^ detected SJ-specific activity not only in the left but also in the right TPJ by measuring more participants [*n* = 14 in Lux *et al*.^12^, *n* = 35 in Hanayik *et al*.^37^. Moreover, the cluster size of the SJ-related activation area was larger for the right TPJ (cluster size: 961) than for the left TPJ (cluster size: 691) [see Table 1 and Fig. 2 in Hanayik *et al*.^37^]. This current evidence suggests the involvement of the right IPL in visual SJ.

For audio-visual simultaneity perception, some neuroimaging studies^11,38–40^ have reported neural correlates in the context of multi-sensory integration. However, the results are not always consistent across studies. The different results among studies are likely due to differences in task settings (e.g., sequential or discrete stimuli, with or without explicit answers). Here, we focus on the results from Bushara *et al*.^11^, who used relatively similar task settings as we did in the present study (e.g., participants judged only one pair of stimuli for each trial and made an explicit answer). The results revealed SJ-specific activity in the right IPL, IFG and anterior insula, and the left cerebellum. Thus, the right IPL was also observed in the audio-visual SJ. In light of previous evidence combined with our result, the right IPL is possibly an essential neural basis of SJs across sensory modalities.

### Regions activated by successive stimuli during SJ

The (SJ_diff_ > SJ_same_) > (NJ_diff_ > NJ_same_) contrast reveals the region specifically activated when successive stimuli were presented during SJ. In this contrast, we observed left-dominant activation in the striatum, anterior insula, pre-SMA, MCG and ACG. These regions were reported as neural correlates of interval timing^7^. In particular, the striatum has been recognized as a core neural substrate for time-interval estimation^1,6^. In our previous fMRI study^9^, the striatum was included in the regions that were commonly activated both tactile SJ and TOJ. Detection of successiveness of two stimuli is necessary not only for SJs but also for the early process of TOJs^16,17^. We infer that the decision of whether two tactile stimuli were successive was made based on the time intervals between the stimuli as estimated by the striatum and the other relevant regions.

Meanwhile, there was no region that exhibited increased activity in the (SJ_same_ > SJ_diff_) > (NJ_same_ > NJ_diff_) contrast. This result suggests that there is no tactile “simultaneity” detector in the brain. That is, the judgment of simultaneity by the brain may be based on non-detection of successiveness. In addition, the right IPL did not exhibit greater activity in the (SJ_same_ > SJ_diff_) > (NJ_same_ > NJ_diff_) contrast nor in the (SJ_diff_ > SJ_same_) > (NJ_diff_ > NJ_same_) contrast. This observation suggests that during SJ, there was no difference in activity of the right IPL between when tactile stimuli were presented successively and simultaneously. That is, the right IPL was activated in response to both successive and simultaneous stimuli during SJ. Accordingly, we infer that the neural process for tactile SJ is as follows: the striatum and the other relevant regions estimate the time interval between tactile stimuli; based on this interval, the right IPL discriminates the successiveness or simultaneity of the stimuli.

### Lack of significant activation of the posterior insula

In our previous fMRI study^9^, compared to tactile TOJ, tactile SJ more strongly activated the left posterior insula. Accordingly, we proposed that the posterior insula plays a role as a detector or comparator of tactile simultaneity. However, the posterior insula did not exhibit greater activity in the SJ > NJ contrast in the present study. Instead, the anterior insula was detected as one of the activated regions in the (SJ_diff_ > SJ_same_) > (NJ_diff_ > NJ_same_) contrast. The anterior insula was reported as one of the neural correlates of audio-visual SJ^11^. In our previous fMRI study^9^ using tactile tasks, however, the anterior insula was detected in the TOJ > SJ contrast under a liberal threshold (cluster 7 in Supplementary Table S1 in Miyazaki *et al*.^9^). At least in tactile SJ, we should treat the anterior insula as a region different from the posterior insula.

To carry out a thorough search, we further analysed the SJ > NJ contrast and the (SJ_diff_ > SJ_same_) > (NJ_diff_ > NJ_same_) contrast, using a liberal threshold at the voxel level (*p* < 0.01 uncorrected, but using *p* < 0.05 FWE corrected at the cluster level) (Supplementary Tables S1–S4). The left posterior insula revealed greater activity in the (SJ_diff_ > SJ_same_) > (NJ_diff_ > NJ_same_) contrasts analysed by SPM (Supplementary Fig. S3 and Table S3) and by SnPM (Supplementary Fig. S4 and Table S4). Meanwhile, no significant greater activity was detected in the (SJ_same_ > SJ_diff_) > (NJ_same_ > NJ_diff_) contrast even using the liberal threshold. Moreover, there was still no significant greater activity in the (NJ_diff_ > NJ_same_) > (SJ_diff_ > SJ_same_) or (NJ_same_ > NJ_diff_) > (SJ_same_ > SJ_diff_) contrast.

The results of the (SJ_diff_ > SJ_same_) > (NJ_diff_ > NJ_same_) contrasts suggest that the left posterior insula was activated in response to successive pairs of tactile stimuli during SJ. In our previous fMRI study^9^, we used −180, −90, −30, −10, −5, 0, +5, +10, +30, +90 and +180 ms for SOAs of tactile stimuli. Meanwhile, the SOAs used in the present study were −50, 0 and +50 ms, appearing in a frequency ratio of 1:2:1. Thus, physically successive stimuli appeared more frequently in the SJ of our previous fMRI study (10/11, i.e., 91%) than in the present study (2/4, i.e., 50%). We therefore infer that SJ-specific activity of the left posterior insula in our previous study was induced by the successive tactile stimuli that actually constituted 91% of trials.

In this context, a question arises. What is the functional difference between the posterior insula and the other regions (e.g., anterior insula, striatum) in the present (SJ_diff_ > SJ_same_) > (NJ_diff_ > NJ_same_) contrast? In our previous fMRI study^9^, the posterior insula was detected in the SJ > TOJ contrast, but the other regions were not. Rather, the anterior insula and striatum exhibited TOJ-related activity. The anterior insula was found in the TOJ > SJ contrast under a liberal threshold. The striatum was included in a common activated area of TOJ and SJ. A possible factor to resolve this question is the vector (i.e., order) of stimuli, which is necessary for TOJ but not for SJ. The posterior insula might be activated in response only to the scalar (i.e., absolute duration) of stimulus asynchrony and not to the vector. In contrast, the anterior insula and the striatum might respond to both. This hypothesis can also account for the relatively lower activity of the posterior insula in the present study, as follows.

Psychophysical^41^ and fMRI^42^ studies reported that the brain adapts to the duration of stimuli. That is, after repetitive exposures to stimuli with an identical duration, neural responses to the stimulus duration are attenuated. When responding only to the absolute stimulus time interval (e.g., SOA = 50 ms) irrespective of the stimulus order (SOA = −50 ms or +50 ms, left or right hand earlier), the posterior insula was more frequently subject to repetitions of the identical stimulus time interval (SOA = 50 ms). The repetitions of the identical time interval could induce neural adaptation to the time interval, resulting in relatively lower activity of the posterior insula.

### Limitations and future perspectives

In the above sections, we first demonstrated that the right IPL is an SJ-specific region, according to the results of the SJ > NJ contrasts. Subsequently, we identified the regions activated by successive stimuli during SJ, according to the results of the interaction contrasts among the tasks (SJ/NJ) and stimulation types (same/difference). At this stage, the results of the interaction contrasts have some possible limitations. Based on the limitations, we further discuss possible concurrent or alternative mechanisms that can account for the results.

Behavioural results were balanced between the tasks. However, taking into account the factor of the stimulation type, the accuracy rates exhibited the lowest average value in the SJ_diff_ condition (Table 2), suggesting that tasks were most difficult under SJ_diff_. In the fMRI analyses, we regressed out the difference in the accuracy rates. However, it should have been optimal to balance the behavioural results among the conditions at the stage of the fMRI recording. For example, the pre-SMA was detected in the (SJ_diff_ > SJ_same_) > (NJ_diff_ > NJ_same_) contrast. The pre-SMA was reported as one of neural correlates of interval timing^7,43^, whereas this region was suggested to be activated related to higher task difficulty of TOJ than SJ in our previous fMRI study^9^. The pre-SMA activity in the present (SJ_diff_ > SJ_same_) > (NJ_diff_ > NJ_same_) contrast might be due to a lingering effect of the high task difficulty in SJ_diff_. To clarify the possible effect of task difficulty in the future, we should control task difficulty for SJ_diff_ (e.g., by controlling SOAs). If including the other task(s) as another control condition, it is so difficult or impossible to clearly balance task difficulty among the conditions. It would be reasonable to focus on differences in neural activity between SJ_diff_ and SJ_same_ in future studies.

Second, in analysing the behavioural results in more detail (see Supplementary Behavioural Results), we found significant effects of SOAs and pin numbers and the significant interactions between them. The results indicate that the participants judged SJ and NJ most accurately when the tactile stimuli were presented with the same timing and same pin numbers (Supplementary Table S5). Thus, “simultaneous” judgements were affected by numerical coincidence of the stimuli, and “same-number” judgements were affected by simultaneity of the stimuli. This result is consistent with a theory of magnitude (ATOM)^27,44^. According to ATOM, various dimensions of magnitude information (e.g., space, time, quantity, numbers) are represented by common neural metrics. The behavioural interaction between time and numbers was also observed in visual TOJ^45^. The (SJ_same_ > SJ_diff_) > (NJ_same_ > NJ_diff_) contrast did not show greater activity in any region in the present study. In the above, we inferred that this result reflects the non-existence of a tactile “simultaneity” detector in the brain. However, this result may be explained by extending the idea of ATOM to perceptual coincident detections. The “simultaneous” and “same-number” detections may share one or more common neural substrates. That is, we could not have been able to find any region specifically activated by simultaneous stimuli during SJ, since we used NJ for the control task in the present study. To test the hypothesis based on ATOM and to clarify the existence/non-existence of the simultaneity detector in the brain, we need future neuroimaging studies using other control tasks that would not share the processing for coincident detection with SJ.

As described thus far, the present fMRI study indicates that the right IPL is a neural correlate of tactile SJ. A similar right parietal area was also detected in audio-visual SJ^11^ and visual SJ^37^. On the other hand, these studies also found SJ-specific activity in the right IFG and anterior insula, and the left cerebellum for audio-visual SJ^11^ and in the bilateral IFG and the left TPJ for visual SJ^37^. The neural activity of audio-visual SJ was detected using a colour judgement with attending to sounds for a control task, and that of visual SJ was detected using visual-orientation and colour judgements for control tasks. Therefore, at this stage, it is not clear whether the differences of the SJ-specific regions among studies were due to differences in sensory modality or that in control tasks. Brain regions detected as those specific to a certain task can vary according to the control tasks. In fact, the left posterior insula was detected in the SJ > TOJ contrast of our previous fMRI study^9^, but it was not detected in the SJ > NJ contrast of the present study. There is only a limited number of neuroimaging studies for SJs, while countless studies on interval timing have been reported^1,4–7^. To consistently elucidate the neural bases of SJs, further neuroimaging studies are still required.

## Summary and Conclusion

In this study, we conducted fMRI to investigate the time-relevant neural correlates of tactile SJ using a time-irrelevant control task (tactile NJ). As our main result, we detected SJ-specific activity in the right TPJ. In this region, the right IPL was detected not only by a standard parametric analysis but also by a novel non-parametric statistical analysis. Moreover, the activation intensity of the right IPL also fulfilled the strict statistical criterion. Accordingly, we demonstrated the right IPL as a time-relevant neural correlate of tactile SJ. In addition, we observed that some left-dominant regions (e.g., the striatum) were specifically activated by successive stimuli during SJ. Meanwhile, there was no region specifically activated by simultaneous stimuli during SJ. This observation suggests that the brain has no simultaneity detector for tactile stimuli but decides “simultaneity” according to non-detection of successiveness. Accordingly, we infer the neural process for tactile SJ to be as follows: the striatum and the other relevant regions (e.g., insula) estimate the time interval between tactile stimuli; based on this interval, the right IPL discriminates the successiveness or simultaneity of the stimuli. Moreover, we further discussed possible concurrent or alternative mechanisms that could explain our fMRI results, taking into account the detailed behavioural results, including the factors of stimulus coincidence (same/different) and features (time/numbers).

## Methods

### Participants

Thirty-two healthy individuals (27 males, 5 females; mean age: 21.9 years; age range: 18–33 years) participated in the present study. We replaced three participants, two because they frequently slept during the tasks and one because the participant exhibited an accuracy rate for NJ of approximately 0.5 (i.e., chance level). Two of the authors of this manuscript were included in the 32 participants. None of the participants had any neurological, psychiatric, or other medical problems at the time of the experiment. All participants were right-handed according to the Edinburgh Handedness Inventory (laterality quotient: 0.90 ± 0.18).

This study was approved by the ethics committee of Kochi University of Technology. All experiments were performed in accordance with the approved guidelines and regulations. All participants provided written informed consent.

### Apparatus

MRI scans were performed using a 3T Siemens Verio scanner equipped with a 32-channel head matrix coil (Siemens, Munich, Germany). Each participant laid in the scanner on their backs wearing headphones (MR-HA01; Kiyohara Optics Inc., Tokyo, Japan). A set of non-magnetic stimulus-response devices (Fig. 1a) was placed in a comfortable position on each participant to deliver tactile stimuli to both index fingers and to allow the participant to make responses with both thumbs.

The stimulus-response set consisted of a pair of Braille stimulators (TI-1101; KGS Corporation, Saitama, Japan) and a pair of response pads (HHSC-2 × 4-D; Current Designs Inc., Philadelphia, USA). The distance between the left and right stimulators was 20 cm. Each stimulator was equipped with 8 movable pins (4 × 2 arrays, inter-pin distance: 3 mm) that were driven by piezoelectric actuators. Tactile stimuli were caused by 2 or 6 pins (Fig. 1b) that protruded 0.7 mm out of the surface of each stimulator. The participants received the stimuli with the ventral surfaces of their index fingers and reported their judgements by pushing buttons on the response pads with their thumbs.

### Stimuli

In each trial, two tactile stimuli were delivered, one to each of the ventral pads of the index fingers (Fig. 1c). The SOAs were −50 ms (left earlier), 0 ms (simultaneous), or +50 ms (right earlier). The appearance ratio of the SOAs was 1:2:1 (i.e., simultaneous and successive stimuli each appeared in 50% of trials). The protruding pins in both stimulators were simultaneously retracted 1000 ms after the onset of pins on the later side. The number of protruded pins in each tactile stimulator was 2 or 6 with two types of pin configurations (Fig. 1b). Accordingly, the difference in the number of pins on each index finger was −4 (left greater), 0 (same), or +4 (right greater). The appearance ratio of the number differences was 1:2:1 (i.e., same- and different-number stimuli each appeared in 50% of trials).

The SOAs and number of protruded pins were selected such that there was no difference in the accuracy rate or reaction time between SJ and NJ, based on the preliminary behavioural measurements (*n* = 10, two of whom also participated in the fMRI experiments). In the fMRI experiments, although there was no difference in the accuracy rate between SJ and NJ, there was a significant difference in the reaction time between the tasks (see *Behavioural Results* above). However, the reaction time was longer in the NJ control task, which is not a problem for interpreting regions associated with SJ in the SJ > NJ contrast (see Davis *et al*.^8^).

### Tasks

In the SJ task, the participants were required to judge whether the onsets of the left and right stimuli were simultaneous. In the NJ task, the participants were required to judge whether the number of pins of the left and right stimuli were the same. During the experiments, the participants closed their eyes.

Half of the participants (*n* = 16) pushed the left button when they judged the left and right stimuli to be simultaneous in SJ or the same number in NJ, whereas they pushed the right button when they judged that the stimuli were successive in SJ or different numbers in NJ. The other half of the participants (*n* = 16) pushed the buttons in the opposite manner.

The participants were instructed to make SJ and NJ in a two-alternative forced choice manner, and they were instructed to push the buttons after the pins’ retractions as accurately and quickly as possible.

### Procedure

During fMRI scanning, each participant completed 4 sessions (2 SJ and 2 NJ sessions). The participants alternated between the SJ and NJ sessions. Half of the participants (*n* = 16) started with an SJ session (i.e., SJ-NJ-SJ-NJ), and the other half (*n* = 16) started with an NJ session (i.e., NJ-SJ-NJ-SJ). Each session consisted of 32 trials, and the inter-trial interval was set at 12.5 s (400 s in total). The 32 trials comprised an equal number of the following four stimulus conditions (timings × numbers): “simultaneous and same”, “simultaneous and different”, “successive and same”, and “successive and different”. These stimulus conditions appeared in a pseudo-random order, and the same stimulus sequences were used for SJ and NJ.

Prior to the fMRI scanning, the participants performed short practice sessions (4 trials/session) for SJ and NJ until they were correct for all trials.

### MRI imaging

Functional images sensitive to blood oxygen level-dependent (BOLD) signals were obtained from a T2* gradient-echo echo-planar imaging pulse sequence with the following parameters: repetition time (TR) = 2500 ms; echo time (TE) = 30 ms; flip angle (FA) = 70°; matrix size = 64 × 64; field of view (FoV) = 200 mm × 200 mm; 39 ascending 3.2 mm thick slices with a 25% slice gap. The functional time series consisted of 175 volumes. After the functional images were acquired, a T1-weighted high-resolution anatomical image was obtained using a magnetization-prepared rapid-acquisition gradient-echo sequence (TR = 2500 ms; TE = 4.32 ms; flip angle = 8°; matrix size = 256 × 256; FoV = 230 mm × 230 mm; voxel size = 0.9 mm × 0.9 mm × 1 mm).

### Behavioural data analysis

Using the response data, we calculated the accuracy rates and reaction times. Reaction times were measured as the time intervals from the timings of the stimulus retractions to those of the onset of the participant responses.

### fMRI data analysis

Image processing and statistical analyses were performed using SPM12 (http://www.fil.ion.ucl.ac.uk/spm) with MATLAB 2013b (MathWorks Inc., Natick, MA, USA). We discarded the first 5 volumes of each fMRI run (i.e., session). To correct for head movement, the functional images were realigned to the first image and again realigned to the mean image after the first realignment, and they were then resliced. Slice-timing correction was conducted on the realigned and resliced functional images, and the T1 anatomical image was co-registered to the mean of the realigned images. The co-registered T1 anatomical image was segmented into tissue class images using a unified segmentation-normalization approach^46^. Subsequently, by using the diffeomorphic anatomical registration through exponentiated Lie algebra (DARTEL) algorithm^47^, the grey and white matter images were transformed to a common coordinate space to create a study-specific template. The study-specific template was then affine normalized to the Montreal Neurological Institute (MNI) space. The parameters of the DARTEL transformation and the affine registration among the study-specific template space and MNI space were applied to spatially normalize each functional image and T1 anatomical image to the MNI space. The resulting functional images were resampled to a voxel size of 2 mm × 2 mm × 2 mm and smoothed using an isotopic Gaussian kernel of 6 mm full width at half-maximum.

We used a random effects model^48^ for the statistical analysis. First, we performed a single-participant analysis using the general linear model (GLM)^49,50^. The task-related neural activity for each condition was modelled using a delta function and convolved with a canonical haemodynamic response function. We used a high-pass filter with a discrete cosine basis function and a cut-off period of 128 s to eliminate artificial low-frequency trends. To reduce motion-related artefacts, 6 realignment parameters estimated in the realignment step were added to the GLM as nuisance regressors. Serial autocorrelation, which assumes a first-order autoregressive model, was estimated using the pooled active voxels with a restricted maximum likelihood procedure and was used to whiten the data and design matrix^51^.

The GLM included the task (SJ or NJ) and the stimulation type (same or difference) as factors of interest. Contrast images were generated for each participant and then entered into a full factorial model to create a random effect SPM{T}. All conditions were modelled according to within-subject levels (i.e., repeated-measures design). Based on our interests, we analysed the main effects of the task (SJ > NJ, NJ > SJ) and the interactions between the task and the stimulation type [(SJ_same_ > SJ_diff_) > (NJ_same_ > NJ_diff_), (SJ_diff_ > SJ_same_) > (NJ_diff_ > NJ_same_), (NJ_same_ > NJ_diff_) > (SJ_same_ > SJ_diff_), (NJ_diff_ > NJ_same_) > (SJ_diff_ > SJ_same_)]. Significantly activated voxels were identified using *p* < 0.001 uncorrected at the voxel level and *p* < 0.05 FWE corrected at the cluster level^25^. To avoid false activations resulting from deactivations in the contrastive conditions, we applied inclusive masks to the analyses as follows. The SJ > NJ contrasts were inclusively masked with the SJ > rest contrast. The NJ > SJ contrast was inclusively masked with the NJ > rest contrast. The (SJ_same_ > SJ_diff_) > (NJ_same_ > NJ_diff_) contrast was inclusively masked with the SJ_same_ > rest and SJ_same_ > SJ_diff_ contrasts. The (SJ_diff_ > SJ_same_) > (NJ_diff_ > NJ_same_) contrast was inclusively masked with the SJ_diff_ > rest and SJ_diff_ > SJ_same_ contrasts. The (NJ_same_ > NJ_diff_) > (SJ_same_ > SJ_diff_) contrast was inclusively masked with the NJ_same_ > rest and NJ_same_ > NJ_diff_ contrasts. The (NJ_diff_ > NJ_same_) > (SJ_diff_ > SJ_same_) was inclusively masked with the NJ_diff_ > rest and NJ_diff_ > NJ_same_ contrasts. For the contrasts used for inclusive masks (e.g., SJ > rest, SJ_diff_ > rest), significantly activated voxels were determined using a default threshold for masking in SPM (*p* < 0.05 uncorrected).

Moreover, we also conducted a non-parametric statistical analysis with SnPM13 toolbox (http://warwick.ac.uk/snpm) to validate the results of the parametric statistical analyses. A recent study^26^ proposed that false positive results possibly occur when using the cluster-level correction for multiple comparisons in standard parametric statistical methods including SPM. Notably, SnPM was developed by an author of the critical report highlighting this issue^26^. Contrasts similar to those in SPM were created by SnPM based on one-sample *t*-tests, since the full factorial model cannot be used in SnPM.

In the SnPM contrasts, we regressed out differences in behavioural results between the tasks or among the conditions as covariates of no interest. We used covariates only in the one-sample *t*-tests, since there are some technical problems in the inclusion of covariates for the factorial model with repeated measures (e.g., Mclaren^52^). The mean reaction time was longer in NJ than in SJ. Therefore, the difference in reaction times between the tasks was regressed out as a covariate of no interest in the SnPM contrasts between tasks. Including a factor of the stimulation type (same/difference), the accuracy rates exhibited the significant main effect of the stimulation type and the interaction between the task and stimulation type. Accordingly, in the interaction contrasts [e.g., (SJ_diff_ > SJ_same_) > (NJ_diff_ > NJ_same_)], we regressed out the corresponding differences in the accuracy rates [e.g., (SJ_diff_ − SJ_same_) − (NJ_diff_ − NJ_same_): −0.10 ± 0.25 (mean ± SD)] as a covariate of no interest. Notably, the differences in the accuracy rates had a significant negative correlation with those in the reaction times [(SJ_diff_ − SJ_same_) − (NJ_diff_ − NJ_same_): 64.9 ± 186.8 ms] (*r* = −0.51, *p* = 0.003); therefore, we used only the difference in the accuracy rates as a covariate in the contrast.

We labelled brain regions using MRIcron (http://www.mricro.com) and Talairach Client (ver. 2.4.3; http://www.talairach.org/client.html)^53^. When using the Talairach Client, we converted MNI coordinates of the peak activations to Talairach coordinates using the icbm2tal transformation (http://www.brainmap.org/icbm2tal/).

## Supplementary information

Supplementary information

## Data Availability

The datasets generated and/or analysed during the current study are available from the corresponding authors on reasonable request.
