# Supplementary material for: Neural correlates of tactile simultaneity judgement: a functional magnetic resonance imaging study
Source: Sci Rep. 2019 Dec 20;9:19481. doi: 10.1038/s41598-019-54323-7 (PMC6925270; doi:10.1038/s41598-019-54323-7)
Supplement: Supplementary file 1 — Supplementary information [file 41598_2019_54323_MOESM1_ESM.docx]

**Supplementary information**

**Neural correlates of tactile simultaneity judgement: a functional magnetic resonance imaging study**

Takahiro Kimura^1, 2*^, Hiroshi Kadota^2, 3^, Tsuyoshi Kuroda^4, 5^, Tomomi D Funai^5^, Makoto Iwata^2, 3^, Takanori Kochiyama^6^, Makoto Miyazaki^4, 5*^

1. Institute of Liberal Arts and Science, Kanazawa University
2. Research Institute, Kochi University of Technology
3. School of Information, Kochi University of Technology
4. Faculty of Informatics, Shizuoka University
5. Research Institute of Time Study, Yamaguchi University
6. ATR Brain Activity Imaging Center

***Corresponding authors:**

**Takahiro Kimura** <kimura@staff.kanazawa-u.ac.jp>

**Makoto Miyazaki** <miyazaki-makoto@inf.shizuoka.ac.jp>

**Supplementary fMRI Results**

**Figure S1.** The brain region that was more strongly activated during simultaneity judgement (SJ) than during number judgement (NJ) (*n* = 32). Activated voxels were identified using SnPM13 with a threshold of *p* < 0.001 uncorrected at the voxel level (*T* > 3.39) and *p* < 0.05 FWE corrected at the cluster level. In this contrast, the difference in reaction times between the tasks was regressed out as a covariate of no interest. Notably, the peak activation in the right IPL also exceeded the significance threshold of *p* < 0.05 FWE corrected at the voxel level (*T* > 5.60). IPL: inferior parietal lobule.

**Figure S2.** Brain regions that were specifically activated by successive stimuli during SJ (*n* = 32). These activated regions were extracted using the (SJ_diff_ > SJ_same_) > (NJ_diff_ > NJ_same_) contrast calculated with SnPM13. SJ_diff_: SJ with successive stimuli, SJ_same_: SJ with simultaneous stimuli, NJ_diff_: NJ with different-number stimuli, NJ_same_: NJ with same-number stimuli. Significantly activated voxels were identified using a threshold of *p* < 0.001 uncorrected at the voxel level (*T* > 3.39) and *p* < 0.05 FWE corrected at the cluster level. In this contrast, the difference in accuracy rates among the conditions was regressed out as a covariate of no interest. SFG: superior frontal gyrus; MCG: middle cingulate gyrus; ACG: anterior cingulate gyrus.

**Figure S3.** Brain regions specifically activated by successive stimuli during SJ (*n* = 32), identified using a liberal statistical threshold at the voxel level. These activated regions were extracted using the (SJ_diff_ > SJ_same_) > (NJ_diff_ > NJ_same_) contrast, calculated with SPM12. Significantly activated voxels were identified using a threshold of *p* < 0.01 uncorrected at the voxel level (*T* > 2.46) and *p* < 0.05 FWE corrected at the cluster level. This contrast was inclusively masked with the SJ_diff_ > rest and SJ_diff_ > SJ_same_ contrasts (*p* < 0.05 uncorrected).

**Figure S4.** Brain regions activated by successive stimuli during SJ (*n* = 32), identified using a liberal statistical threshold at the voxel level. These activated regions were extracted using the (SJ_diff_ > SJ_same_) > (NJ_diff_ > NJ_same_) contrast, calculated with SnPM13. Significantly activated voxels were identified using a threshold of *p* < 0.01 uncorrected at the voxel level (*T* > 2.36) and *p* < 0.05 FWE corrected at the cluster level. In this contrast, the difference in accuracy rates among the conditions was regressed out as a covariate of no interest.

**Table S1**. Activated clusters in the SJ > NJ contrast, calculated with SPM12 using a liberal statistical threshold at voxel level (*p* < 0.01 uncorrected at the voxel level; *p* < 0.05 FWE corrected at the cluster level). This contrast was inclusively masked with the SJ > rest contrast (*p* < 0.05 uncorrected). * denotes that the activation level exceeded the threshold of *p <* 0.05 FWE corrected at the voxel level (*T* > 5.00). IPL: inferior parietal lobule; SMG: supramarginal gyrus; MTG: middle temporal gyrus; STG: superior temporal gyrus; ACG: anterior cingulate gyrus; MedFG: medial frontal gyrus; IFG: inferior frontal gyrus; MFG: middle frontal gyrus.

|  |  | MNI coordinates | | |  |  |  |
| --- | --- | --- | --- | --- | --- | --- | --- |
| Cluster # | Size (voxels) | x | y | z | *T*_124_ | L/R | Region |
| 1 | 1243 | 58 | -48 | 42 | 5.52* | R | IPL |
|  |  | 60 | -44 | 28 | 3.70 | R | SMG |
|  |  | 54 | -16 | -16 | 3.44 | R | MTG |
|  |  | 58 | -42 | 16 | 3.39 | R | STG |
| 2 | 1583 | 8 | 46 | 0 | 4.08 | R | ACG/MedFG |
|  |  | 0 | 46 | 18 | 3.86 |  | MedFG/ACG |
|  |  | -4 | 50 | -6 | 2.92 | L | MedFG/ACG |
| 3 | 777 | 42 | 36 | -12 | 3.42 | R | IFG |
|  |  | 48 | 22 | 2 | 3.28 | R | IFG |
|  |  | 36 | 50 | -6 | 3.00 | R | MFG |

**Table S2**. Activated cluster in the SJ > NJ contrast, calculated with SnPM13 using a liberal statistical threshold at the voxel level (*p* < 0.01 uncorrected at the voxel level; *p* < 0.05 FWE corrected at the cluster level). In this contrast, the difference in reaction times between the tasks was regressed out as a covariate of no interest. * denotes that the activation level exceeded the threshold of *p <* 0.05 FWE corrected at the voxel level (*T* > 5.65). IPL: inferior parietal lobule; MTG: middle temporal gyrus; STG: superior temporal gyrus; SMG: supramarginal gyrus.

|  |  | MNI coordinates | | |  |  |  |
| --- | --- | --- | --- | --- | --- | --- | --- |
| Cluster # | Size (voxels) | x | y | z | *T*_30_ | L/R | Region |
| 1 | 1471 | 56 | -48 | 42 | 5.79* | R | IPL |
|  |  | 54 | -18 | -14 | 4.33 | R | MTG |
|  |  | 60 | -48 | 20 | 3.58 | R | STG/SMG |

**Table S3**. Activated clusters in the (SJ_diff_ > SJ_same_) > (NJ_diff_ > NJ_same_) contrast, calculated with SPM12 using a liberal statistical threshold at the voxel level (*p* < 0.01 uncorrected at the voxel level; *p* < 0.05 FWE corrected at the cluster level). This contrast was inclusively masked with the SJ_diff_ > rest and SJ_diff_ > SJ_same_ contrasts (*p* < 0.05 uncorrected). SFG: superior frontal gyrus; MCG: middle cingulate gyrus; ACG: anterior cingulate gyrus; MedFG: medial frontal gyrus.

|  |  | MNI coordinates | | |  |  |  |
| --- | --- | --- | --- | --- | --- | --- | --- |
| Cluster # | Size (voxels) | x | y | z | *T*_124_ | L/R | Region |
| 1 | 3102 | -4 | -8 | 0 | 4.69 | L | Thalamus |
|  |  | 12 | 6 | 14 | 4.04 | R | Caudate |
|  |  | -20 | 12 | 2 | 4.03 | L | Putamen |
|  |  | 26 | 2 | 10 | 3.93 | R | Putamen |
|  |  | -38 | 0 | 2 | 3.87 | L | Posterior Insula |
|  |  | 8 | -6 | -4 | 3.68 | R | Thalamus |
|  |  | -30 | 20 | 6 | 3.52 | L | Anterior Insula |
|  |  | -16 | 16 | 4 | 3.38 | L | Caudate |
| 2 | 1190 | -6 | 26 | 40 | 3.81 | L | SFG/MCG |
|  |  | 10 | 26 | 32 | 3.45 | R | MCG |
|  |  | -8 | 30 | 28 | 3.42 | L | ACG |
|  |  | -10 | 18 | 50 | 3.39 | L | SFG |
|  |  | 8 | 28 | 46 | 2.96 | R | SFG/MedFG |

**Table S4**. Activated clusters in the (SJ_diff_ > SJ_same_) > (NJ_diff_ > NJ_same_) contrast, calculated with SnPM13 using a liberal statistical threshold at the voxel level (*p* < 0.01 uncorrected at the voxel level; *p* < 0.05 FWE corrected at the cluster level). In this contrast, the difference in accuracy rates among the conditions was regressed out as a covariate of no interest. MFG: middle frontal gyrus; IFG: inferior frontal gyrus; SFG: superior frontal gyrus; ACG: anterior cingulate gyrus; MCG: middle cingulate gyrus.

|  |  | MNI coordinates | | |  |  |  |
| --- | --- | --- | --- | --- | --- | --- | --- |
| Cluster # | Size (voxels) | x | y | z | *T*_30_ | L/R | Region |
| 1 | 2400 | 26 | 24 | -8 | 5.10 | R | Anterior Insula |
|  |  | 44 | 10 | 40 | 4.75 | R | MFG |
|  |  | 12 | 6 | 14 | 4.57 | R | Caudate |
|  |  | 14 | -12 | 4 | 4.53 | R | Thalamus |
|  |  | 50 | 20 | 6 | 4.38 | R | IFG |
| 2 | 1860 | -10 | 18 | 50 | 4.97 | L | SFG |
|  |  | -6 | 28 | 32 | 4.76 | L | ACG/MCG |
|  |  | 8 | 32 | 30 | 4.36 | R | ACG |
|  |  | 4 | 16 | 48 | 4.01 | R | SFG |
| 3 | 2095 | -22 | 12 | 2 | 4.93 | L | Putamen |
|  |  | -54 | 28 | 22 | 4.46 | L | MFG/IFG |
|  |  | -28 | 22 | 6 | 4.43 | L | Anterior Insula |
|  |  | -58 | 16 | 20 | 4.21 | L | IFG |
|  |  | -38 | 0 | 2 | 3.88 | L | Posterior Insula |
|  |  | -16 | 16 | 4 | 3.75 | L | Caudate |

**Supplementary Behavioural Results**

*Accuracy rate (task × stimulus timing × stimulus number)*

Table S5 shows the accuracy rates across the participants, calculated for each task [simultaneity judgement (SJ) or number judgement (NJ)], each stimulus onset asynchrony (SOA) between the tactile stimuli [0 ms (simultaneous), −50 ms (left earlier), or +50 ms (right earlier)] and each combination of the numbers of pins used for the tactile stimuli [*N*_pins_: 2 vs. 2, 6 vs. 6, 2 vs. 6, or 6 vs. 2 (left vs. right)]. We conducted a three-way repeated-measures analysis of variance (ANOVA) (2 tasks × 3 SOAs × 4 *N*_pins_) on the accuracy rates.

In summary, the results of the three-way ANOVA supported the results regarding accuracy rates in the main body of the paper. Moreover, the results newly revealed that “simultaneous” judgements were affected by numerical coincidence of the stimuli, and “same-number” judgements were affected by temporal coincidence of the stimuli. The details are as follows.

The ANOVA indicated no significant main effect of task [*F*(1, 31) = 0.45, *p* = 0.51, *η_p_*^2^ = 0.014], further supporting no difference in the accuracy rates between SJ and NJ that was shown by the *t*-test to compare the accuracy rates for SJ and NJ (see *Comparison between tasks* in **Behavioural results** and Table 1 in the main body of the paper).

Meanwhile, the ANOVA indicated significant main effects of SOA [*F*(2, 62) = 12.79, *p* < 0.001, *η_p_*^2^ = 0.29] and *N*_pins_ [*F*(3, 93) = 3.24, *p* = 0.026, *η_p_*^2^ = 0.095] and the interaction between them [*F*(6, 186) = 2.96, *p* = 0.009, *η_p_*^2^ = 0.087]. The ANOVA also indicated a significant interaction between task and SOA [*F*(2, 62) = 7.37, *p* = 0.001, *η_p_*^2^ = 0.19]. However, there was no significant interaction between task and *N*_pins_ [*F*(3, 93) = 2.21, *p* = 0.092, *η_p_*^2^ = 0.067] or among the three factors [*F*(6, 186) = 0.71, *p* = 0.64, *η_p_*^2^ = 0.023].

The analyses of simple main effects for the interaction between SOA and *N*_pins_ revealed that the effect of SOA was significant at the *N*_pins_ values of 2 vs. 2 [*F*(2, 62) = 20.43, *p* < 0.001, *η_p_*^2^ = 0.40] and 6 vs. 6 [*F*(2, 62) = 8.53, *p* < 0.001, *η_p_*^2^ = 0.22]. Multiple comparisons using Holm’s method indicated that, at the *N*_pins_ values of 2 vs. 2 and 6 vs. 6, the accuracy rates for the SOA of 0 ms were significantly higher than those for the SOAs of −50 ms and +50 ms (*p* ≤ 0.004, Cohen’s *d* ≥ 0.60). The analyses of simple main effects also revealed that the effect of the *N*_pins_ was significant at the SOA of 0 ms [*F*(3, 93) = 8.34, *p* < 0.001, *η_p_*^2^ = 0.21]. Multiple comparisons using Holm’s method indicated that, at an SOA of 0 ms, the accuracy rates for the *N*_pins_ of 2 vs. 2 and 6 vs. 6 were significantly higher than those for the *N*_pins_ of 2 vs. 6 and 6 vs. 2 (*p* ≤ 0.034, *d* ≥ 0.48). For the rest, there was no significant effect in the analyses of simple main effects (*p* ≥ 0.12, *η_p_*^2^ ≤ 0.061) and no significant difference in the other multiple comparisons (*p* ≥ 0.18, *d* ≤ 0.24).

Thus, these results regarding the interaction between the SOA and *N*_pins_ showed that the participants judged SJ and NJ most accurately when the tactile stimuli were presented with same timing and same pin numbers. That is, “simultaneous” judgements were affected by numerical coincidence of the stimuli, and “same-number” judgements were affected by temporal coincidence of the stimuli.

The analyses of simple main effects for the interaction between task and SOA revealed that the effect of task was significant at the SOA of 0 ms [*F*(1, 31) = 6.39, *p* = 0.017, *η_p_*^2^ = 0.17] and that the effect of SOA was significant in SJ [*F*(2, 62) = 12.63, *p* < 0.001, *η_p_*^2^ = 0.29]. Multiple comparisons using Holm’s method indicated that in SJ, the accuracy rate for the SOA of 0 ms was significantly higher than those of −50 ms and +50 ms (*p* ≤ 0.002, *d* ≥ 0.64), but there was no difference between those of −50 ms and +50 ms (*p* = 0.39, *d* = 0.15). For the rest, there was no significant effect in the analyses of simple main effects (*p* ≥ 0.10, *η_p_*^2^ ≤ 0.083).

These results regarding the interaction between task and SOA are consistent with those of the two-way ANOVA indicating that the effect of stimulation type (same/different) was significant in SJ but did not reach significance in NJ (see *Comparisons among tasks and stimulation types* in **Behavioural results** and Table 2 in the main body of the paper). These results suggested that the effects of stimulation type on the accuracy rates were more evident for SJ than for NJ. Accordingly, in the interaction contrasts among the tasks and stimulation types, we regressed out the corresponding differences in the accuracy rates among the conditions as covariates of no interest (for details, see **fMRI data analysis** in the **Methods** section of the main body of the paper).

**Table S5**. Accuracy rates [mean (standard deviation)] across the participants (task × timing × number). SJ: simultaneity judgement; NJ: number judgement; SOA: stimulus onset asynchrony; *N*_pins_: combination of numbers of pins for the tactile stimuli (left vs. right).

|  |  | Task | |
| --- | --- | --- | --- |
| SOA | *N*_pins_ | SJ | NJ |
| 0 ms | 2 vs. 2 | 0.96  (0.09) | 0.92  (0.11) |
|  | 6 vs. 6 | 0.98  (0.06) | 0.90  (0.19) |
|  | 2 vs. 6 | 0.90  (0.17) | 0.79  (0.20) |
|  | 6 vs. 2 | 0.88  (0.21) | 0.82  (0.27) |
| −50 ms | 2 vs. 2 | 0.77  (0.24) | 0.84  (0.26) |
|  | 6 vs. 6 | 0.84  (0.23) | 0.88  (0.24) |
|  | 2 vs. 6 | 0.83  (0.18) | 0.84  (0.25) |
|  | 6 vs. 2 | 0.75  (0.25) | 0.92  (0.15) |
| +50 ms | 2 vs. 2 | 0.72  (0.28) | 0.81  (0.25) |
|  | 6 vs. 6 | 0.85  (0.19) | 0.84  (0.24) |
|  | 2 vs. 6 | 0.77  (0.24) | 0.82  (0.20) |
|  | 6 vs. 2 | 0.75  (0.27) | 0.84  (0.19) |

*Reaction time (task × stimulus timing × stimulus number)*

Table S6 shows the reaction times across the participants, calculated for each task (SJ or NJ), each SOA (0 ms, −50 ms, or +50 ms) and each *N*_pins_ (2 vs. 2, 6 vs. 6, 2 vs. 6, or 6 vs. 2). We conducted a three-way repeated-measures ANOVA (2 tasks × 3 SOAs × 4 *N*_pins_) on the reaction times.

In summary, the results of the three-way ANOVA supported those of the reaction times in the main body of the paper. The results were also generally consistent with the above results of the three-way ANOVA on the accuracy rates, although they were unsystematic compared to those of the accuracy rates. The details are as follows.

The ANOVA revealed a significant main effect of task [*F*(1, 31) = 10.78, *p* = 0.003, *η_p_*^2^ = 0.26]. This result is consistent with the result of the *t*-test indicating a significantly longer reaction time for NJ than for SJ (see *Comparison between tasks* in **Behavioural results** and Table 1 in the main body of the paper).

The ANOVA also revealed a significant main effect of SOA [*F*(2, 62) = 7.90, *p* < 0.001, *η_p_*^2^ = 0.20] but not of *N*_pins_ [*F*(3, 93) = 0.18, *p* = 0.91, *η_p_*^2^ = 0.006]. There was a significant interaction between SOA and *N*_pins_ [*F*(6, 186) = 2.62, *p* = 0.019, *η_p_*^2^ = 0.078] but not between task and SOA [*F*(2, 62) = 1.58, *p* = 0.21, *η_p_*^2^ = 0.049], between task and *N*_pins_ [*F*(3, 93) = 2.51, *p* = 0.064, *η_p_*^2^ = 0.075], or among the three factors [*F*(6, 186) = 0.79, *p* = 0.58, *η_p_*^2^ = 0.025].

The analyses of simple main effects for the interaction between the SOA × *N*_pins_ revealed that the effect of SOA was significant at the *N*_pins_ of 2 vs. 2 [*F*(2, 62) = 10.63, *p* < 0.001, *η_p_*^2^ = 0.26] and 6 vs. 6 [*F*(2, 62) = 4.38, *p* < 0.017, *η_p_*^2^ = 0.12]. Multiple comparisons using Holm’s method indicated that in the *N*_pins_ of 2 vs. 2, the reaction times for the SOA of 0 ms were significantly shorter than those for the SOAs of −50 ms and +50 ms (*p* = 0.0014, *d* = 0.69). In the *N*_pins_ of 6 vs. 6, the reaction times were significantly shorter for the SOAs of 0 ms and −50 ms than for +50 ms (*p* ≤ 0.035, *d* ≥ 0.44). The analyses of simple main effects also indicated that the effect of the *N*_pins_ was significant at a SOA of −50 ms [*F*(3, 93) = 3.13, *p* = 0.030, *η_p_*^2^ = 0.092]. Multiple comparisons using Holm’s method indicated that, at a SOA of −50 ms, the reaction time for the *N*_pins_ value of 2 vs. 2 was longer than those for the *N*_pins_ values of 6 vs. 6 and 2 vs. 6 (*p* ≤ 0.045, *d* ≥ 0.49). For the rest, there was no other significant effect in the analyses of simple main effects (*p* ≥ 0.19, *η_p_*^2^ ≤ 0.051) and no significant difference in the multiple comparisons (*p* ≥ 0.27, *d* ≤ 0.34).

Thus, these results regarding the interaction between the SOA and *N*_pins_ showed that the participants judged SJ and NJ consistently in a short time (i.e., readily) when the tactile stimuli were presented with same timing and same pin numbers. Thus, the reaction times were generally similar to the accuracy rates in the interaction between stimulus timing and number, although the effect was unsystematic for the reaction times compared to the accuracy rates.

**Table S6**. Reaction times [mean (standard deviation); unit: ms] across the participants (task × stimulus timing × stimulus number). SJ: simultaneity judgement; NJ: number judgement; SOA: stimulus onset asynchrony; *N*_pins_: combination of numbers of pins for the tactile stimuli (left vs. right).

|  |  | Task | |
| --- | --- | --- | --- |
| SOA | *N*_pins_ | SJ | NJ |
| 0 ms | 2 vs. 2 | 740.7  (271.7) | 877.2  (258.7) |
|  | 6 vs. 6 | 784.2  (390.9) | 914.1  (366.6) |
|  | 2 vs. 6 | 905.3  (518.9) | 867.5  (335.9) |
|  | 6 vs. 2 | 848.8  (340.4) | 878.6  (301.3) |
| −50 ms | 2 vs. 2 | 869.7  (318.8) | 1008.8  (359.7) |
|  | 6 vs. 6 | 762.8  (231.2) | 943.7  (300.5) |
|  | 2 vs. 6 | 793.1  (289.3) | 919.6  (343.5) |
|  | 6 vs. 2 | 846.7  (313.9) | 891.8  (362.8) |
| +50 ms | 2 vs. 2 | 858.9  (311.7) | 983.8  (321.3) |
|  | 6 vs. 6 | 867.2  (390.4) | 1014.2  (375.7) |
|  | 2 vs. 6 | 894.2  (359.1) | 963.9  (327.9) |
|  | 6 vs. 2 | 848.7  (342.5) | 928.9  (377.2) |
